# Delay Kalman Filter to Estimate the Attitude of a Mobile Object with Indoor Magnetic Field Gradients

**DOI:** 10.3390/mi7050079

**Published:** 2016-05-02

**Authors:** Christophe Combettes, Valérie Renaudin

**Affiliations:** GEOLOC laboratory, The French Institute of Science and Technology for Transport, Spatial Planning, Development and Networks (IFSTTAR), Route de Bouaye CS4, 44344 Bouguenais Cedex, France; christophe.combettes@ifsttar.fr

**Keywords:** attitude estimation, extended Kalman filter, Kalman filter with delay, inertial mobile unit (IMU), magnetometer, pedestrian navigation

## Abstract

More and more services are based on knowing the location of pedestrians equipped with connected objects (smartphones, smartwatches, *etc*.). One part of the location estimation process is attitude estimation. Many algorithms have been proposed but they principally target open space areas where the local magnetic field equals the Earth’s field. Unfortunately, this approach is impossible indoors, where the use of magnetometer arrays or magnetic field gradients has been proposed. However, current approaches omit the impact of past state estimates on the current orientation estimate, especially when a reference field is computed over a sliding window. A novel Delay Kalman filter is proposed in this paper to integrate this time correlation: the Delay MAGYQ. Experimental assessment, conducted in a motion lab with a handheld inertial and magnetic mobile unit, shows that the novel filter better estimates the Euler angles of the handheld device with an 11.7° mean error on the yaw angle as compared to 16.4° with a common Additive Extended Kalman filter.

## 1. Introduction

Many smart connected objects have been developed for the internet of things (IoT). They are meant to facilitate daily life activities with many services that are based on embedded sensors. Among these services are geolocation of lost objects, home automation services or monitoring human well-being. These sensors are primarily made of telecommunication chipsets and inertial micro electro-mechanical sensors (MEMS): a tri-axis accelerometer and tri-axis gyroscope. Tri-axis magnetometers are more and more available in connected objects. Indeed, whereas several years ago, hardware manufacturers had to mount separated triads of accelerometers and gyroscopes on printed circuit boards, they are now commonly available in a single 9-degrees-of-freedom magnetic and inertial mobile unit (MIMU), which consists of calibrated and co-aligned triads of accelerometers, gyroscopes and magnetometers. As they are increasingly available in everyday objects, there is an increasing interest in the development of methods to process the magnetometer measurements and propose novel IoT personal applications.

**Figure 1:**
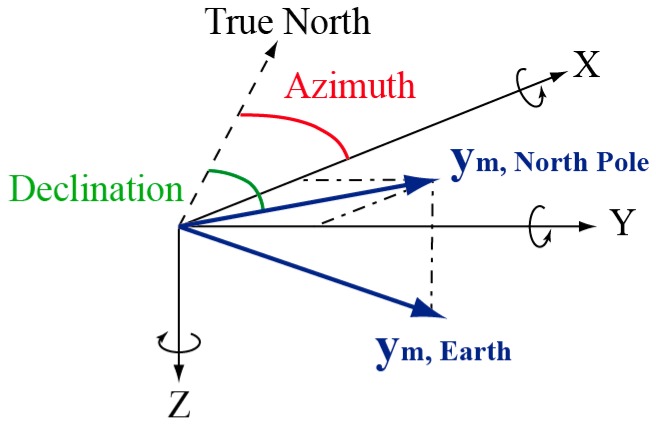
Estimation of the azimuth angle using the Earth’s magnetic field measurement $y_{m}$ and the declination angle between the True North and the horizontal component.

Historically, magnetometer and compass records were processed to estimate the direction toward the Earth North Magnetic Pole. As illustrated in Figure 1, this direction is changed into the azimuth angle, *i.e.*, angular direction toward the True (geographic) North, using the local deviation angle extracted from geographical tables. This popular method can only be applied if the measured magnetic field vector corresponds to the Earth Magnetic Field. Indeed, when the magnetometer measurement is perturbed, the estimated direction points toward the local magnetic perturbation source instead of the North Pole [1]. Consequently, this method is only valid in environments where there is no artificial source of magnetic field. This is typically the case in open outdoor spaces, but not in the urban and indoor spaces where most IoT applications are expected to function. To overcome this limitation, research has been conducted to invent novel processing strategies of magnetic field measurements for urban/indoor surroundings. Three main different approaches have been proposed to process magnetometer data even when they are perturbed by artificial sources of magnetic field:

1. Magnetic field fingerprinting (FP);
2. Velocity estimation from spatial magnetic field gradients measured with an assembly of magnetometers;
3. Attitude angles estimation from magnetic field gradients measured in time with a single tri-axis magnetometer.

### 1.1. Magnetic Field Fingerprinting (FP)

The first approach is based on the fingerprinting principle that was first developed to geolocate mobile objects using Wi-Fi receiver signal strength (RSS) data. In the context of magnetometer based positioning, instead of using Wi-Fi RSS, magnetic field amplitudes are used [2]. The method works as follows. In an offline phase, there is a map of indoor/urban magnetic field amplitudes. A database stores all amplitude fingerprints along with their geographical coordinates in the map. During the online phase, real time magnetic field measurements are compared with the database to extract the associated geographical positions.

The advantage of this method is that it directly provides the user’s location in the mapped environment and it is built on the diversity and complexity of indoor/urban magnetic fields to distinguish between different geographical positions. Similarly to Wi-Fi FP, this approach assumes that the measured urban/indoor magnetic fields are the same as the one measured to build the map in the offline phase. This is a strong assumption that is not always true. It is the for example the case when the smart object is close to an elevator lift or during emergency situations when all metal doors are automatically closed to prevent the spread of fire. In these situations, the performance will deteriorate.

### 1.2. Velocity Estimation from Spatial Magnetic Field Gradients Measured with an Assembly of Magnetometers

The second approach uses an array of magnetometers separated by known distances to measure the local spatial magnetic field gradient and derive the velocity estimate. The magnetic field space gradient is related to the derivative of the position and the magnetic field temporal derivate based on Biot-Savart laws [3]. A recursive process is then applied to track the mobile object in time using the successive velocity estimates and knowing the initial geographical coordinates of the magnetometer array.

The performance of this method strongly depends on the calibration of each individual magnetometer because they must all measure the same magnetic field, which can be very challenging when the hosting platform exhibits varying fields. The distance between each magnetometer is also critical to precisely measure the spatial gradient. Globally, the performance of this method increase in surroundings with large magnetic field anomalies, and the best performances are achieved in environments where many artificial sources of magnetic field are present. Because the magnitude of the magnetic field rapidly drops when the separation between the artificial field source and the magnetometer array increases (it follows an inverse cubic law), on many occasions the use of gradients is not sufficient to compute good velocity estimates and complementary methods are needed.

### 1.3. Attitude Angles Estimation from Magnetic Field Gradients Measured in Time by a Single Tri-Axis Magnetometer

The third approach computes the magnetic field gradient based on magnetometer measurements that are recorded at different epochs while the mobile object is moving. When the local magnetic field is assessed as static over two consecutive epochs, it is used to observe the rotation of the mobile object between the two epochs. The angular rate of the dynamic object is then derived from the successive quasi static magnetic field records [4]. Then, a recursive process estimates the attitude angles of the connected object using the angular rate estimates and the known initial attitude angles. In this approach requires only one tri-axis magnetometer.

When the local magnetic field is fluctuating over time (e.g., power on of an IT object, a moving elevator, *etc.*), this approach cannot be applied. To mitigate this issue, magnetically derived angular rates are merged with gyroscopes angular rates and accelerations. This hybridization enables continuous tracking of the orientation of the MIMU. It provides also a continuous calibration of the large accelerometer and gyroscope errors that are inherent to the low cost nature of MEMS. Existing attitude estimation filters are principally based on the Extended Kalman Filter (EKF) with a quaternion parameterization of the attitude angles. Quaternion parameterization has also been recently proposed to model the gyroscope signal [5]. Three popular hybridization filters have been tested for manual and locomotion tasks performed by six human subjects [6]. A comparison of four main hybridization filters of MIMU signals has also been recently conducted in a motion laboratory [7]. Despite good performances (several degrees in average), they show that magnetic anomalies are still a challenge for many filters.

### 1.4. Challenge of Attitude Estimation Filters Based on Magnetic Fields Recorded at Different Epochs

Existing filters, which are based on the magnetically derived angular rate principle, merge magnetic field data that have been recorded at different epochs: at the beginning of the quasi static magnetic field period (*t*_0_) and at the epoch of state’s computation (*t_k_*). However they do not consider the fact that the orientation of the mobile object is different at the two epochs 0 and *k*, which limits the solution. They also do not consider the correlation between the two epochs and the quality of the orientation estimate at the beginning of the quasi static period. This approximation is made in existing filters where averaged data and instantaneous data are fused to estimate the attitude at epoch *k*.

These limitations are addressed in this article with a novel delay EKF, which is called the Delay Magnetic, Acceleration Fields and Gyroscope Quaternion (Delay MAGYQ) and considers the states’ correlation over the quasi-static period of magnetic field. The novelty is that this algorithm considers the quality of the magnetic field estimated at the beginning of the quasi static period, which serves as a reference for deriving the angular rates, and its correlation with the current epoch to estimate the orientation angles of the mobile object and its variance. The past MAGYQ filter does not consider this time correlation to estimate the attitude angles.

## 2. The Delay MAGYQ Attitude Estimation Filter

### 2.1. MEMS Signals Modelling

A signal model is introduced for each accelerometer, gyroscope and magnetometer measurement. These mathematical models are essential to represent the measurement errors that are known to be very large with MEMS and must be mitigated in the attitude estimation process.

#### 2.1.1. Magnetometer Signal Model

The tri-axis magnetometer senses the local magnetic field vector. In order to measure only the surrounding magnetic field and not the one produced by the hosting platform, it must first be calibrated. Soft and hard iron errors are estimated along with other deterministic errors due to the fabrication process [8,9]. Once the magnetometer is calibrated, it can be modeled by:

(1) $$y_{m}=m^{b}+n_{m}$$

where $y_{m}$ is the magnetometer measurement, $m^{b}$ is the local magnetic field expressed in the body frame (frame of mobile object) and $n_{m}$ is a white Gaussian noise (0, $\sigma_{m}^{2}$).

#### 2.1.2. Accelerometer Signal Model

The accelerometer measures the specific force experienced by the IMU. Its output is a vector that can be modeled by:

(2) $$y_{a}=a_{ib}^{b}-g^{b}+b_{a}+n_{a}$$

where $y_{a}$ is the measured acceleration vector, $a_{ib}^{b}$ is the acceleration vector expressed in the body frame with respect to the inertial frame, $n_{a}$ is a white Gaussian noise (0, $\sigma_{a}^{2}$) and $b_{a}$ is the accelerometer bias.

#### 2.1.3. Gyroscope Signal Model

The gyroscope measures the angular rate of the MIMU in the body frame with respect to the inertial frame and some error terms. The gyroscope signal is modeled by:

(3) $$y_{g}=\omega_{ib}^{b}+b_{g}+n_{g}$$

where $y_{g}$ is the gyroscope measurement, $\omega_{ib}^{b}$ is the angular rate of the MIMU with respect to the inertial frame, expressed in the body frame, $n_{g}$ is a white Gaussian noise (0, $\sigma_{g}^{2}$) and $b_{g}$ is the gyroscope bias.

Instead of considering the gyroscope’s signal as an angular rate, it can be expressed in terms of rotational elements. The exponential function of the quaternion is used to perform the change of state as follows:

(4) $$\forall q\in ℍ,\exp\left(q\right)=\sum_{i=0}^{+\infty }\frac{{\left(q\right)}^{i}}{i!}$$

The average angular velocity ω between two epochs, corresponding to the time interval Δ*t*, induces the rotation $q_{\omega ,\Delta t}$ defined by $q_{\omega ,\Delta t}=\exp\left(\frac{\Delta t}{2}{\left(\omega \right)}_{q}\right)$, with ${\left(\omega \right)}_{q}={\left(\begin{array}{cc} 0 & \omega^{T} \end{array}\right)}^{T}$ the quaternion form of a vector. Based on the quaternion set, a new gyroscope signal model is proposed:

(5) $$q_{y_{g},\Delta t}=q_{\omega ,\Delta t}+b_{q_{g},\Delta t}+n_{q_{g},\Delta t}$$

$q_{y_{g},\Delta t}$ is the quaternion corresponding to the rotation sensed by the gyroscope between two consecutive epochs over the time interval Δ*t*. $q_{\omega ,\Delta t}$ is the quaternion that represents the true rotation of the MIMU gyroscope between two successive epochs over the time interval Δ*t*. $n_{q_{g},\Delta t}$ is a white Gaussian noise (0, $\sigma_{q_{g},\Delta t}^{2}$) and $b_{q_{g},\Delta t}$ is the rotation bias introduced by the gyroscope.

## 3. Analysis of the Influence of Past Estimates on the Orientation Estimation with Magnetometer Measurements

### 3.1. Problem Statement

As introduced earlier, the first algorithms, which assume that the magnetometer records correspond to the Earth magnetic field, cannot be applied in urban and indoor surroundings. Indeed, the large time and space fluctuations of the magnetic field in these surroundings complicate the processing of magnetometer data. The goal is still to estimate the attitude angles of the MIMU in the magnetically perturbed environment. This is still possible using magnetic anomalies since they provide useful information about the orientation changes of the MIMU, especially when the local magnetic field is steady over a time interval. This period is the “quasi static field” (QSF) period and it is defined by:

(6) $$T_{QSF}={\left(t_{k}\right)}_{0\leq k\leq N}\text{ and }m_{ref}^{n}$$

where $m_{ref}^{n}$ is the steady magnetic field that is the reference vector for the period. The following observation equation is used to estimate the orientation error using the magnetometer data over the QSF interval.

(7) $${\left(\delta m_{t_{k}}^{n}\right)}_{q}={\left(m_{ref}^{n}\right)}_{q}-{\hat{q}}_{bt_{k}}^{n}⊗{\left(y_{m}\left(t_{k}\right)\right)}_{q}⊗{\bar{\hat{q}}}_{bt_{k}}^{n}$$

To estimate $\delta m_{t_{k}}^{n}$ in Equation (7), the reference magnetic field $m_{ref}^{n}$ must first be computed. It is defined at the beginning of the quasi static period $T_{QSF}$ from the magnetometer measurement at epoch $t_{0}$ by:

(8) $${\left({\hat{m}}_{ref_{T_{QSF}}}^{n}\right)}_{q}={\hat{q}}_{bt_{0}}^{n}⊗{\left(y_{m}\left(t_{0}\right)\right)}_{q}⊗{\bar{\hat{q}}}_{bt_{0}}^{n}$$

The reference magnetic field in Equation (8) is estimated from the measurements and the orientation estimate introducing two different errors. The first error comes from the magnetometer measurement that is associated to a white Gaussian noise. This error can be mitigated with a low pass filter. The second error comes from the orientation estimation of the MIMU at epoch $t_{0}$, which is not perfect.

Knowing the reference magnetic field from Equation (8), it is possible to estimate the innovation $\delta z_{k}$, at epoch $t_{k}$ from Equation (7), which becomes:

(9) $${\left(\delta z_{t_{k}}\right)}_{q}={\left({\hat{m}}_{ref_{T_{QSF}}}^{n}\right)}_{q}-{\hat{q}}_{bt_{k}}^{n}⊗{\left(y_{m}\left(t_{k}\right)\right)}_{q}⊗{\bar{\hat{q}}}_{bt_{k}}^{n}$$

### 3.2. Magnetic Field Based Observation Equation

Equation (9) is expanded using the reference field $m_{ref}^{n}$ associated to the QSF period.

(10) $${\left(\delta z_{t_{k}}\right)}_{q}={\left({\hat{m}}_{ref_{T_{QSF}}}^{n}\right)}_{q}-{\left(m_{ref}^{n}\right)}_{q}+{\left(m_{ref}^{n}\right)}_{q}-{\hat{q}}_{bt_{k}}^{n}⊗{\left(y_{m}\left(t_{k}\right)\right)}_{q}⊗{\bar{\hat{q}}}_{bt_{k}}^{n}$$

This equation is linked to two error terms. The first one $\delta m_{t_{k}}^{n}$ comes from the transformation of the magnetic field measured in the body frame into the navigation frame at epoch $t_{k}$. The navigation frame corresponds to the local mapping frame (East North Up frame). The second error term $\delta m_{t_{0}}^{n}$ comes from the transformation of the magnetic field measured in the body frame into the navigation frame at epoch $t_{0}$. This second error term also corresponds to the error on the reference magnetic field estimate over the quasi-static period.

(11) $$\delta z_{k}=\delta m_{k}^{n}-\delta m_{0}^{n}$$

The first error, which comes from the transformation of the magnetic field into the navigation frame $\delta m_{t_{k}}^{n}$, can be related with a first order development to the error on the quaternion estimate $\delta q_{bt_{k}}^{n}$by:

(12) $$\delta m_{k}^{n}=2\left[\begin{array}{cc} A_{t_{k}} & a_{t_{k}}Id-\left[A_{t_{k}}\times \right] \end{array}\right]\delta q_{bt_{k}}^{n}+C_{b}^{n}\left({\hat{q}}_{bt_{k}}^{n}\right)n_{m}$$

where $a_{t_{k}}=\langle {\hat{q}}_{bt_{k}}^{n},{\left({\hat{m}}_{t_{k}}^{n}\right)}_{q}\rangle$ is the scalar product, $A_{t_{k}}=q_{1}{}_{t_{k}}{\hat{m}}_{t_{k}}^{n}-\left[u_{q}{}_{t_{k}}\times \right]{\hat{m}}_{t_{k}}^{n}$ with ${\hat{q}}_{bt_{k}}^{n}={\left(\begin{array}{cc} q_{1}{}_{t_{k}} & u_{qt_{k}} \end{array}\right)}^{T}$, $C_{b}^{n}\left({\hat{q}}_{bt_{k}}^{n}\right)$ is the rotation matrix calculated from the quaternion estimate ${\hat{q}}_{bt_{k}}^{n}$, $\left[y\times \right]=\left(\begin{array}{ccc} 0 & -y_{3} & y_{2}\\ y_{3} & 0 & -y_{1}\\ -y_{2} & y_{1} & 0 \end{array}\right)$ is the antisymmetric matrix of the vector $y={\left(\begin{array}{ccc} y_{1} & y_{2} & y_{3} \end{array}\right)}^{T}$.

The innovation $\delta z_{t_{k}}$, which is sensed by the magnetometer, can be related to the errors on the orientation estimation $\delta q_{bt_{k}}^{n}$ and $\delta q_{bt_{0}}^{n}$ by

(13) $$\delta z_{k}=H_{t_{k}}\delta q_{bt_{k}}^{n}-H_{t_{0}}\delta q_{bt_{0}}^{n}+M_{t_{k},t_{0}}n_{m}$$

The Jacobean matrices in Equation (13) are given by:

(14) $$H_{t_{k}}=2\left[\begin{array}{cc} A_{t_{k}} & a_{t_{k}}Id-\left[A_{t_{k}}\times \right] \end{array}\right],\;H_{t_{0}}=2\left[\begin{array}{cc} A_{t_{0}} & a_{t_{0}}Id-\left[A_{t_{0}}\times \right] \end{array}\right]$$

and

(15) $$M_{t_{k},t_{0}}=\left[C_{b}^{n}\left({\hat{q}}_{bt_{k}}^{n}\right)+C_{b}^{n}\left({\hat{q}}_{bt_{0}}^{n}\right)\right]$$

## 4. Design of the Novel Attitude Estimation Filter: Delay MAGYQ

The design of the novel attitude estimation filter Delay MAGYQ is based on [5]. The modification to integrate the delay induced by the use of an averaged angular rate is inspired by [10]. Other articles have also addressed the delay approach in Kalman Filter [11,12].

### 4.1. State Vector

The eleven parameters state vector is given by

(16) $$x={\left[\begin{array}{ccc} q_{b}^{n} & b_{q_{g},\Delta t} & b_{a} \end{array}\right]}^{T}\in {\mathbb{R}}^{11}$$

where $q_{b}^{n}$ is the quaternion that describes the transformation of the MIMU between the body and the navigation frames, $b_{a}$ is the accelerometer bias introduced in Equation (2) and $b_{q_{g},\Delta t}$ is the gyroscope quaternion bias given in Equation (5).

### 4.2. Dynamic Evolution of the State Vector

The rotation $q_{\omega t_{k-1},\Delta t}$ between the epochs $t_{k-1}$ and $t_{k}$ is first estimated. The subscript Δ*t* is omitted in the development of the following equations to ease the reading. ${\hat{x}}_{t_{k}}={\left[\begin{array}{ccc} {\hat{q}}_{bt_{k}}^{n} & {\hat{b}}_{q_{g}t_{k}} & {\hat{b}}_{at_{k}} \end{array}\right]}^{T}$ is the state vector estimate at epoch $t_{k}$.

(17) $$\{\begin{array}{c} q_{y_{g}t_{{}_{k-1}}}=\exp\left({\left(y_{gt_{{}_{k-1}}}\right)}_{q}\right)\\ {\hat{q}}_{\omega t_{k-1}}=q_{y_{g}t_{{}_{k-1}}}-{\hat{b}}_{q_{g}t_{k-1}}\\ {\hat{q}}_{\omega t_{{}_{k-1}}}=\frac{{\hat{q}}_{\omega t_{{}_{k-1}}}}{\left\|{\hat{q}}_{\omega t_{{}_{k-1}}}\right\|} \end{array}$$

The quaternion $q_{b}^{n}$ is propagated using the gyroscope measurements. The biases $b_{q_{g}}$ and $b_{a}$ are modeled with random walks.

(18) $$\left\{ \begin{array}{l} {\hat{q}}_{bt_{k}}^{n}={\hat{q}}_{bt_{k-1}}^{n}⊗{\hat{q}}_{\omega t_{k-1}}\\ {\hat{b}}_{q_{g}t_{k}}={\hat{b}}_{q_{g}t_{k-1}}\\ {\hat{b}}_{at_{k}}={\hat{b}}_{at_{k-1}} \end{array}\right.$$

### 4.3. Dynamic Evolution of the Covariance Matrix

The covariance matrix $P_{t_{k}}$ is associated to the perturbation of state vector $\delta x_{t_{k}}$. The time evolution of $P_{t_{k}}$ depends on the stochastic models chosen to describe the evolution in time of the biases $b_{q_{g}}$ and $b_{a}$. This choice impacts the Jacobian matrices $F_{t_{k-1}}$ and $G_{t_{k-1}}$ and the covariance matrix $Q_{t_{k-1}}$ associated with the system noise. They are given by

(19) $$\left\{ \begin{array}{l} F_{t_{k-1}}=\left(\begin{array}{ccc} C_{{\hat{q}}_{\omega }t_{{}_{k-1}}} & -M_{{\hat{q}}_{bt_{{}_{k-1}}}^{n}} & 0\\ 0 & I & 0\\ 0 & 0 & I \end{array}\right)\\ G_{t_{k-1}}=\left(\begin{array}{ccc} -M_{{\hat{q}}_{bt_{{}_{k-1}}}^{n}} & 0 & 0\\ 0 & T_{s}I & 0\\ 0 & 0 & T_{s}I \end{array}\right) \end{array}\right.\Rightarrow P_{t_{k}}=F_{t_{k-1}}P_{t_{k-1}}F_{t_{k-1}}{}^{T}+G_{t_{k-1}}Q_{t_{k-1}}G_{t_{k-1}}{}^{T}$$

The matrices $M_{q}$ and $C_{q}$ are defined using the quaternion product, which is modified into a product of matrices by

(20) $$q_{1}⊗q_{2}=M_{q_{1}}q_{2}=C_{q_{2}}q_{1}$$

### 4.4 Time Evolution of the Covariance Matrix Involving Past Orientation Estimate

It is assumed that the orientation information sensed by the magnetometer depends on the attitude angles at epoch *t_0_*. This orientation is labelled $q_{bt_{0}}^{n}$. The corresponding estimation error $\delta q_{bt_{0}}^{n}$ is linked to the covariance matrix $P_{q,t_{0}}$. To integrate the time correlation of orientation estimates, the covariance matrix that relates $\delta q_{bt_{0}}^{n}$ and $\delta x_{t_{k}}$ must be computed. It corresponds to the correlation between past orientation estimate and the present one and is given by

(21) $$P_{t_{0}}=\left[\begin{array}{cc} P_{q,t_{0}} & P_{\left(q,b\right),t_{0}}\\ P_{\left(q,b\right),t_{0}}{}^{T} & P_{b,t_{0}} \end{array}\right]$$

with $P_{q,t_{0}}$ is the covariance matrix of $\delta q_{bt_{0}}^{n}$, $P_{b,t_{0}}$ is the covariance matrices of the biases and $P_{\left(q,b\right),t_{0}}$ is the covariance matrix between $\delta q_{bt_{0}}^{n}$ and the biases.

We define also the product $\Gamma_{t_{0},t_{k}}=\prod_{p=0}^{k}F_{t_{p-1}}$ and ${\overset{˘}{P}}_{t_{k}}$ the covariance matrix of the state vector perturbation ${\left[\begin{array}{cc} \delta x_{t_{k}}{}^{T} & \delta q_{bt_{0}}^{n}{}^{T} \end{array}\right]}^{T}$. Using previously introduced notations, this covariance becomes:

(22) $${\overset{˘}{P}}_{t_{k}}=\left[\begin{array}{cc} P_{t_{k}} & \Gamma_{t_{0},t_{k}}\left[\begin{array}{c} P_{q,t_{0}}\\ P_{\left(q,b\right),t_{0}}{}^{T} \end{array}\right]\\ \left[\begin{array}{cc} P_{q,t_{0}} & P_{\left(q,b\right),t_{0}} \end{array}\right]\Gamma_{t_{0},t_{k}}{}^{T} & P_{q,t_{0}} \end{array}\right]$$

### 4.5. Update Equations

The Kalman Gain ${\overset{˘}{K}}_{t_{k}}$ is computed for ${\left[\begin{array}{cc} \delta x_{t_{k}}{}^{T} & \delta q_{bt_{0}}^{n}{}^{T} \end{array}\right]}^{T}$ using Equations (13) and (22).

(23) $${\overset{˘}{K}}_{t_{k}}={\overset{˘}{P}}_{t_{k}}{\overset{˘}{H}}_{t_{k}}{\overset{˘}{S}}_{t_{k}}{}^{-1}$$

where ${\overset{˘}{H}}_{t_{k}}=\left[\begin{array}{ccc} H_{t_{k}} & 0 & -H_{t_{0}} \end{array}\right]$, ${\overset{˘}{S}}_{t_{k}}={\overset{˘}{H}}_{t_{k}}{\overset{˘}{P}}_{t_{k}}{\overset{˘}{H}}_{t_{k}}{}^{T}+M_{t_{k},t_{0}}R_{m}M_{t_{k},t_{0}}{}^{T}$ with $R_{m}$ being the magnetometer measurement covariance matrix.

The error state estimate $\delta x_{t_{k}}$ is updated by:

(24) $$\delta x_{t_{k}}=K_{t_{k}}\delta z_{t_{k}}$$

where ${\overset{˘}{K}}_{t_{k}}=\left[\begin{array}{c} K_{t_{k}}\\ K_{q,t_{0}} \end{array}\right]$.

The update of the global covariance matrix ${\overset{˘}{P}}_{t_{k}}$ is classically computed:

(25) $${\overset{˘}{P}}_{t_{k}}={\overset{˘}{P}}_{t_{k}}-{\overset{˘}{K}}_{t_{k}}{\overset{˘}{H}}_{t_{k}}{\overset{˘}{P}}_{t_{k}}$$

The update of the covariance matrix $P_{t_{k}}$ is performed:

(26) $$P_{t_{k}}=\left(Id-K_{t_{k}}\left[\begin{array}{cc} H_{t_{k}} & 0 \end{array}\right]\right)P_{t_{k}}+K_{t_{k}}H_{t_{0}}\left[\begin{array}{cc} P_{q,t_{0}} & P_{\left(q,b\right),t_{0}} \end{array}\right]\Gamma_{t_{0},t_{k}}{}^{T}$$

## 5. Performance Evaluation with Experimental Data

### 5.1. Experimental Setup

For the experimentation, the subjects hold an MIMU in hand. Two units were used: the ADIS-16488 from Analog Device and the VN-300 from VectorNav (Dallas, TX, USA). Both units comprise a tri-axis gyroscope, a tri-axis accelerometer and a tri-axis magnetometer whose MEMS technical specifications are given in [13,14] respectively. The acquisition frequency is set to 200 Hz for the VN-300 and 100 Hz for the ADIS-16488. 

**Figure 2:**
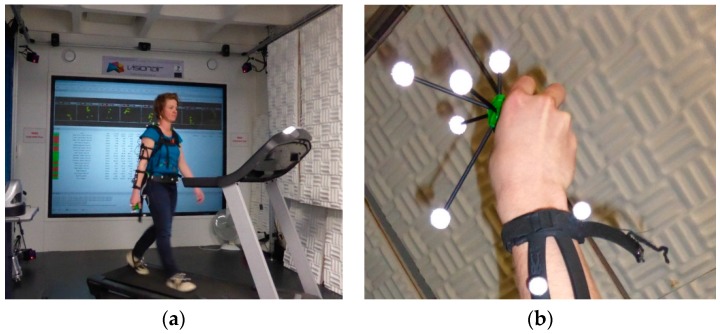
(**a**) Motion capture room equipped with the ART IR tracking system, the treadmill and the test subject holding the MIMU in hand. (**b**) An ART MoCap targets tree is rigidly fixed to the handheld MIMU.

The reference orientations and positions are estimated by the ART IR tracking system [15] installed in a motion capture room (Figure 2a). It consists of 4 cameras on the floor and 4 cameras on the ceiling working at a 60 Hz sampling frequency. The subject is equipped with the ART MoCap target set comprising 6-degrees-of-freedom passive reflective markers. A marker tree is rigidly attached to the handheld MIMU (Figure 2b) in order to track its reference positions and orientations in time. The accuracy of the angular estimation of the ART IT tracking system is about one degree.

The surface of the motion capture room is 9 m^2^, which is too small for performing pedestrian walks. Consequently the test subjects were asked to walk on a treadmill even if this may modify the human walking gait as compared to the natural gait of a pedestrian walking on a non-moving ground. Four test subjects (S1, S2, S3 and S4) participated in the experiment.

### 5.2. Experimental Scenarios

Three different walking modes were performed: “texting”, “swinging” and unsupervised walking. They correspond to the motions that travelers would do in the context of pedestrian navigation using a connected object. All four subjects performed the texting and swinging scenarios whereas only one subject (S1) performed the unsupervised walking scenario.

- Texting mode: The test subjects are walking on the treadmill at a 5 km/h comfortable with handheld IMU in a fixed position as compared to the pedestrian’s center of mass. This position corresponds to a traveler that is reading navigation instructions given on the screen of the connected object. Each dataset includes 100 to 140 strides, which corresponds to approximately 120 s.
- Swinging mode: The walking speed is again set to 5 km/h but the arm is naturally oscillating during the walk. The MIMU is still carried in hand during the acquisition and its duration is the same as in the texting scenario.
- Unsupervised walking: The walking speed ranges between 1 and 1.7 m/s. MIMU data was acquired over a 13 min walk, which corresponds to a 780 m to 1.3 km range of distances. No specific instruction was given to the test subject on how to carry the handheld MIMU. He/she walks freely on the treadmill, simulating the use of a connected object that gives navigation indications. During the experiment, several object carrying modes are observed. They correspond to the outcomes of the human activity classification algorithm defined in [16]. Among them are the “Swinging” mode (natural arm oscillation), the “Texting” mode (the upper limbs are constrained), the “Phoning” mode or the Irregular mode (unclassified).

**Figure 3:**
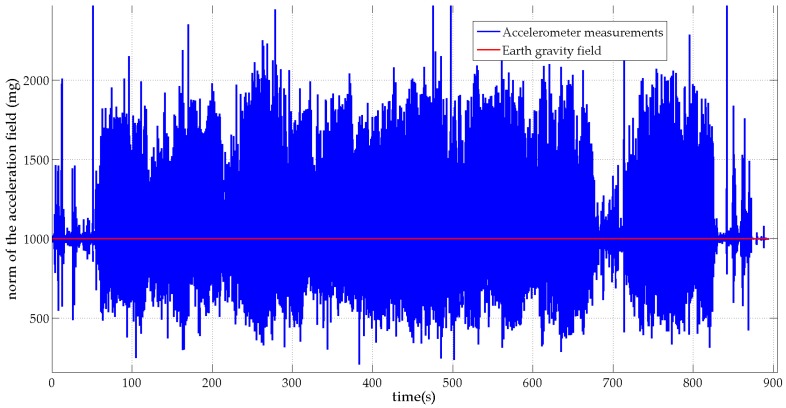
Norm of the MIMU’s acceleration vector (blue) and the Earth gravity field (red).

**Figure 4:**
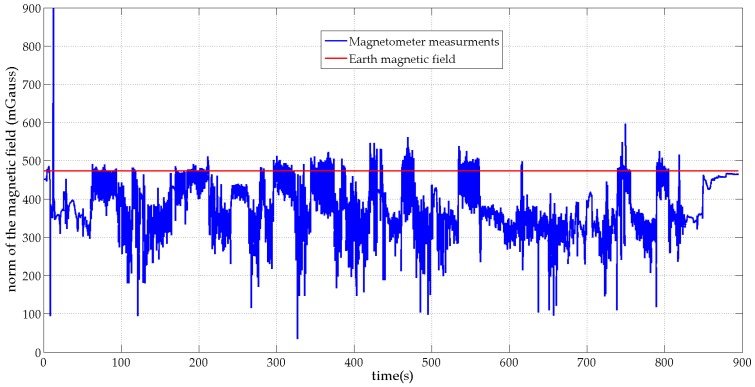
Norm of the local magnetic field and the reference Earth magnetic field.

Figure 3 and Figure 4 show, in blue, the norms of the MIMU’s acceleration vector and the sensed local magnetic field for the unsupervised scenario respectively. For comparison purposes, the norms of the Earth gravity field and the Earth magnetic field are plotted in red on the same figures. Figure 3 shows that the subject performed large-amplitude hand motions during the experiment. Figure 4 shows that many artificial sources of magnetic field are present in the motion lab room with a strongly perturbed Earth magnetic field.

### 5.3. Attitude Estimation Algorithms Comparison Approach for AEKF, MAGYQ and Delay MAGYQ

The MIMU dataset are post-processed with three different attitude estimation algorithms: AEKF, MAGYQ and MAGYQ Delay.

- Additive Extended Kalman Filter. The AEKF is a well-known algorithm that estimates the orientation with a quaternion parameterization in its additive form [17]. It assumes that the tri-axis magnetometer measures the Earth magnetic field. This hypothesis is common in most of existing algorithms [18,19].
- Magnetic, Acceleration Fields and Gyroscope Quaternion (MAGYQ) Based Attitude Estimation. This processing exploits steady magnetic field, even perturbed by artificial sources, to estimate the orientation angles. The reference field is not anymore the Earth field but the magnetometer vector measured at the beginning of the quasi static field period. Magnetic angular rates are then derived. Furthermore this algorithm proposes a gyroscope error modelling directly in the quaternion space to reduce linearization errors [5].
- Delay MAGYQ that is proposed in this paper and consider the correlation between the orientation estimate at the beginning of the quasi static magnetic field period and the orientation estimate at the epoch of the filter’s calculation.

Three different features are used to compare these algorithms:

- The **convergence**, which is used to assess the filter’s ability to correctly estimate the state parameters.
- The **convergence** **speed,** which completes the convergence criteria by including the time needed to achieve the convergence.
- The **stability** for assessing the filter’s ability to continue to correctly estimate the state parameters once the convergence is achieved.

The choice of the initial state parameters impacts the filter’s behavior. The filter’s convergence is more easily assessed when the initial state is different from the “true” values. When the filter is initialized with a state vector that is close to the correct values, it is rather the filter’s stability that is assessed. In what follows, all algorithms are initialized using the outcomes of the ART motion tracking system. This allows concentration on assessing the ability of the filter to correctly estimate the state parameters when large hand motions and large magnetic field fluctuations occur. The accelerometer and magnetometer noises are set to be 10% of the signal to noise ratio. The gyroscope noise is set according to the datasheet, around 0.0035°/(s·Hz^1/2^).

### 5.4. Experimental Results

The performance analysis is conducted on the Euler angles estimates. The angular errors for the roll, pitch and yaw angles are computed using the reference orientations computed by the ART IR tracking system.

#### 5.4.1. Texting Scenario

Table 1 gives the angular errors for all four subjects in the texting scenario. μ corresponds to the absolute value of the mean error and σ is the associated standard deviation.

**Figure 5:**
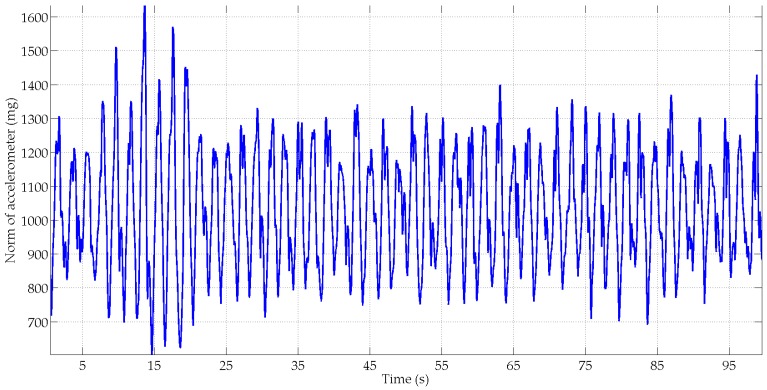
Illustration of the accelerometer’s norm for subject S1 in the texting scenario.

**Figure 6:**
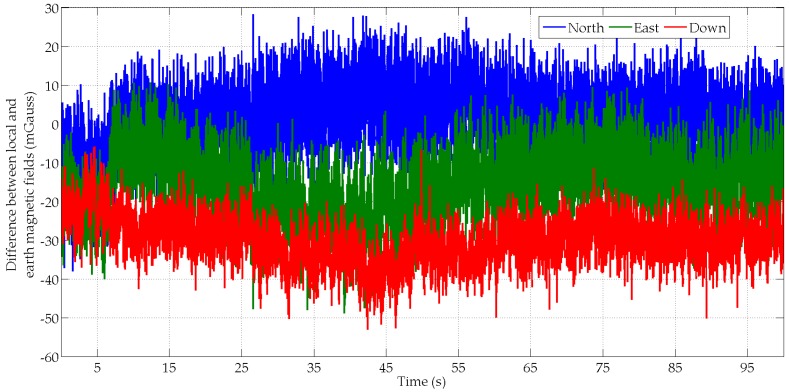
Difference between two magnetic field vectors, *i.e.*, the Earth field and the MIMU’s local field, for the subject S1 in the texting scenario.

The results are similar for all subjects and algorithms, with not more than a 1° difference between the three algorithms. In the texting scenario on the treadmill at 5 km/h, the MIMU’s acceleration is relatively small compared to the Earth’s gravity field (Figure 5). Consequently, the measured accelerations give a good observation of the gravity field in the MIMU’s frame and the roll and pitch angles are well estimated by all algorithms. The yaw angle is also correctly estimated with a 6.2° to 6.7° average error. This is explained by the fact that the local magnetic field is relatively stable and close to the Earth’s magnetic field. This is can be seen in Figure 6 that show the differences between the Earth magnetic field vector and the local magnetic field vector computed with the MIMU’s records that have been transformed into the local frame using the MotionLab orientation data. In this texting scenario, the good magnetic field conditions and the rather small range of MIMU motions explain the good accuracy of the orientation estimates with all algorithms.

#### 5.4.2. Swinging Scenario

Table 2 gives the angular errors for all four subjects in the swinging scenario. Similarly to the texting scenario, μ corresponds to the absolute value of the mean error and σ is the corresponding standard deviation.

**Figure 7:**
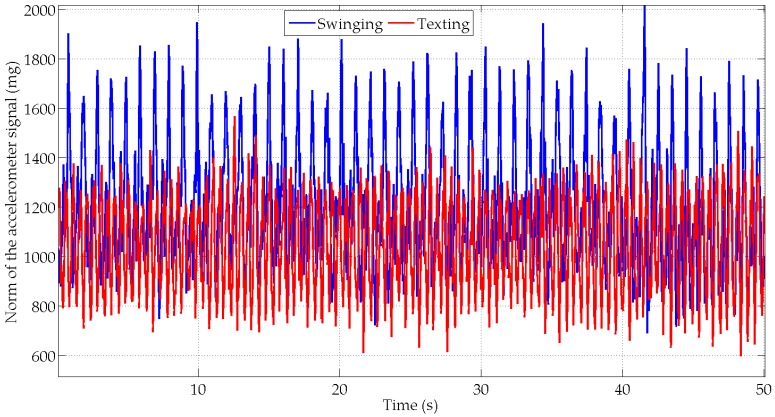
Norm of the accelerations for subject S1 in the texting (red) and swinging (blue) scenarios.

In the swinging scenario, the orientation estimation performance is reduced for all algorithms as compared to texting scenario. The hand movements, which are synchronized with the oscillations of the arm, induce large accelerations as compared to the amplitude of the Earth’s gravity field (Figure 7). These accelerations strongly impact the roll and pitch angles estimation. Consequently the average error is increased by 2° to 4° for these two angles. The drop in performance is even greater on the standard deviation that increases by approximately 10° for the AEKF and MAGYQ filter.

**Figure 8:**
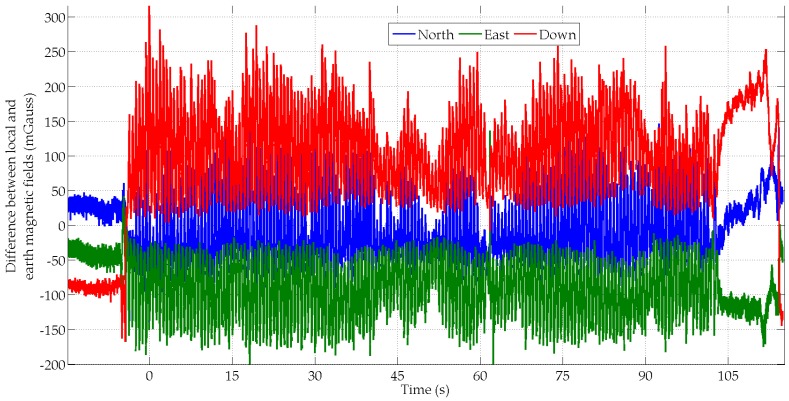
Difference between two magnetic field vectors, *i.e.*, the Earth field and the MIMU’s field, for subject S4 in the swinging scenario.

Large magnetic field variations are observed for all four subjects. This phenomenon is illustrated in Figure 8 for the subject S4. These magnetic field fluctuations especially occur during the arm oscillations when the MIMU gets closer and further apart from the surrounding ferromagnetic compounds. This explains why the accuracy of the estimated yaw angles is fairly poor.

However, the three algorithms have different performances. Delay-MAGYQ achieves better angle estimates with smaller mean errors and smaller standard deviations. Contrary to the MAGYQ filter, Delay–MAGYQ integrates the covariance data of the orientation estimates to compute the reference field, which improves the orientation estimation. Indeed the two filters will process the measurement error in different ways. With the algorithm MAGYQ, the measurement error is completely passed onto the attitude estimate at the current time, whereas with Delay-MAGYQ, it is distributed not only on the estimate at the current time but also on the orientation at the first epoch of the quasi-static phase.

#### 5.4.3. Unsupervised Walking Scenario

**Figure 9:**
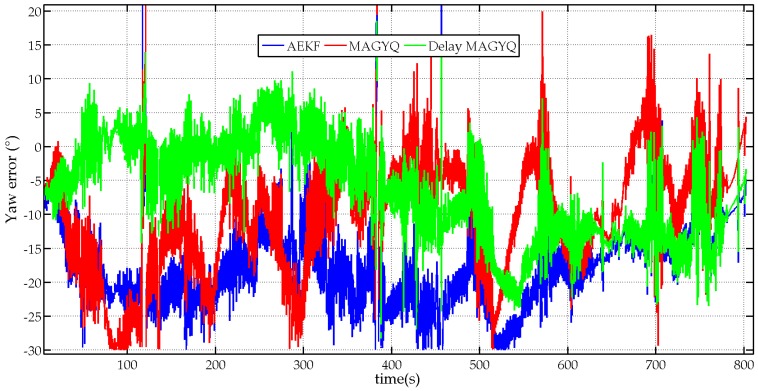
Error on the yaw angle estimates for the three attitude estimation filters: AEKF (bleu), MAGYQ (red) and Delay MAGYQ (green).

The orientation estimation errors and the corresponding statistics are detailed in Table 3 for the unsupervised walking scenario. The yaw estimates for the AEKF, MAGYQ and Delay MAGYQ filters are also plotted in Figure 9 for comparison purposes.

It is observed that the best orientation estimates are achieved with the filter Delay MAGYQ whereas the AEKF gives the worst results. Indeed, the errors on the roll and pitch angles are in the same order of magnitude for both algorithms but the yaw estimate is strongly deteriorated with the AEKF. This is explained by the experimental environment where large magnetic field anomalies are present. The motion acquisition room is equipped with many electronic devices, wires and metallic walls that strongly modify the Earth magnetic field. This can be observed in Figure 2 where the Earth magnetic field norm and the measured local magnetic field norm are plotted in red and blue respectively. Consequently, the AEKF functioning hypothesis *H*_0_, which assumes that the magnetometer measures the Earth magnetic field, is not valid and the orientation gets biased.

MAGYQ and Delay-MAGYQ algorithms are immune to local perturbations because the assumption *H*_0_ is not required to process the raw inertial signals and magnetometer measurements. Consequently, better yaw estimates are obtained thanks to the magnetically derived angular rates with a reference magnetic field that is closer to the reality, *i.e.*, the field at the beginning of the quasi static period. Furthermore the use of magnetic field gradients, when the local field is steady, enables continuous calibration of the gyroscope errors.

Because the Delay MAGYQ algorithm considers the impact of past orientation estimates on the magnetic reference field estimate and the current orientation estimate, it gives the best results with a slightly larger standard deviation. With the Pedestrian Dead Reckoning (PDR) approach, which is applied to provide navigation services on connected objects, the accuracy of the yaw angle directly impacts the accuracy of the geolocation services, whereas roll and pitch angles have a smaller impact on the location estimate. Indeed, current PDR position is derived from the previous position knowing the yaw angle and the step length. This highlights why it is important to consider the correlation between states estimated at different times to enhance the accuracy of the yaw angle and provide more accurate handheld based navigation services.

**Figure 10:**
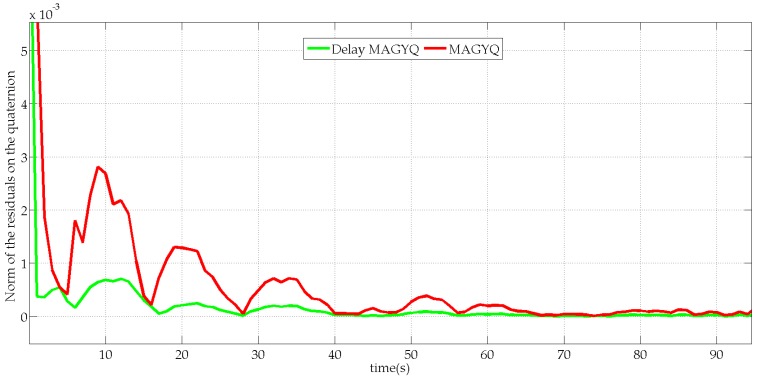
Norm of the residual errors (Kδz) on the quaternion orientation estimated with MAGYQ (red) and Delay-MAGYQ (green).

An innovation test is performed to analyze the gain of Delay-MAGYQ as compared to the MAGYQ filter. The test is performed over a QSF period of magnetic field during which the MIMU is static. The initial orientation is set as equal to the orientation (the reference) estimated by the MotionLab for both filters. The performance of the two filters is analyzed with the norm of the orientation’s residuals: $\delta q_{bt}^{n}$. Figure 10 shows the residual errors estimated by both filters. 

The main observation is that the norm of the innovation is lower for Delay MAGYQ than for MAGYQ. Because the initial orientation of both filters corresponds to the “true” one and there is no orientation change, the filter that exhibits the lowest innovation will perform better. This is observed with Delay MAGYQ, which includes covariance data of the first QSF state’s estimate in the computation of the Kalman gain. Indeed, MAGYQ filter gain is classically given by

(27) $$K_{t_{k}}=P_{t_{k}}H_{t_{k}}{}^{T}{\left(H_{t_{k}}P_{t_{k}}H_{t_{k}}{}^{T}+R_{m}\right)}^{-1}$$

whereas the gain of Delay MAGYQ filter integrates the orientation error $\delta q_{b}^{n}$ in

(28) $$K_{t_{k}}=P_{t_{k}}H_{t_{k}}{}^{T}{\overset{˘}{S}}_{t_{k}}{}^{-1}-\Gamma_{t_{0},t_{k}}\left[\begin{array}{c} P_{q,t_{0}}\\ P_{\left(q,b\right),t_{0}}{}^{T} \end{array}\right]H_{t_{0}}{}^{T}{\overset{˘}{S}}_{t_{k}}{}^{-1}$$

$P_{q,t_{0}}$, $P_{\left(q,b\right),t_{0}}$, ${\overset{˘}{S}}_{t_{k}}$ and $\Gamma_{t_{0},t_{k}}$ are detailed in Equations (21) and (23).

## 6. Conclusions

Whereas some MIMU-based attitude estimation filters try to mitigate the impact of an artificial field on the angular estimation, other exploit the distorted field when the perturbation is assessed as static. They are based on magnetic field gradients and a reference field, which is estimated at the beginning of the period of interest. However, these filters do not consider the time correlation of past state estimates on the current orientation estimate.

A novel Delay MAGYQ filter is proposed to overcome this limitation. The design of the filter directly integrates the time correlation of the orientation estimates, in particular with a covariance matrix that relates the error on the quaternion estimates at previous times with the current state estimate. The impact of including the time correlation in the angles estimation is assessed in a motion laboratory with four persons. Three scenarios are built for the context of pedestrian navigation with a handheld MIMU. They correspond to a total of 30 min of data collection.

The novel filter is found to better estimate the Euler angles of the handheld device. Looking at the yaw angle, it is estimated with an 11.7° mean error by Delay-MAGYQ as compared to a 16.4° with the Additive Extended Kalman filter and a 12.7° mean error without considering the time correlation of the fil**t**er’s estimates. These mean errors are computed for the entire data collection. The same standard deviation, ranging from 7.7° to 8°, is associated to the error on the yaw angle for all three filters. Another finding is that the linear acceleration sensed by the MIMU perturbs the orientation estimation for all filters. Globally, Delay-MAGYQ is found to be more robust thanks to an improved processing of errors at past epochs for the orientation estimates.

## 7. Perspectives

Despite the good performances achieved for the orientation estimation, the identification of the periods during which the local magnetic field is assessed as steady should be improved in order to better apply opportune QSF updates. Being able to mitigate large accelerations in the orientation estimation is also planned in future research, especially when the arm is oscillating during natural walking (e.g., with a smartwatch).

## Figures and Tables

**Table 1:** Angular errors of AEKF, MAGYQ and Delay MAGYQ attitude estimation filters for the texting scenario with all four subjects.

Algorithms	AEKF		MAGYQ		Delay MAGYQ	
Mean/standard deviation	μ	σ	μ	σ	μ	σ
Roll (°)	0.6	0.5	1.8	1.1	1.0	0.9
Pitch (°)	1.8	0.6	2.7	1.8	1.6	1.3
Yaw (°)	6.7	4.7	6.5	4.4	6.2	4.7

**Table 2:** Angular errors of AEKF, MAGYQ and Delay MAGYQ attitude estimation filters for the swinging scenario with all four subjects.

Algorithms	AEKF		MAGYQ		Delay MAGYQ	
Mean/standard deviation	μ	σ	μ	σ	μ	σ
Roll (°)	5.3	14.0	5.0	12.0	3.2	5.3
Pitch (°)	3.8	6.0	3.8	5.0	4.1	5.1
Yaw (°)	24.4	10.6	21.3	9.3	19.0	8.6

**Table 3:** Orientation estimates and their statistics for the unsupervised walking scenario.

Algorithms	AEKF		MAGYQ		Delay MAGYQ	
Mean/standard deviation	μ	σ	μ	σ	*Μ*	σ
Roll (°)	5.8	7.4	6.0	8.8	5.0	8.5
Pitch (°)	2.0	2.6	3.9	4.2	3.3	3.3
Yaw (°)	18.1	8.6	11.3	10.2	8.2	9.8
